# Momentary associations between perceived stress, dietary restriction, and eating behaviors in adolescents

**DOI:** 10.1093/jpepsy/jsag046

**Published:** 2026-07-11

**Authors:** Peichen Liu, Jeremy C Morales, Genevieve F Dunton, Tyler B Mason

**Affiliations:** Suzanne Dworak-Peck School of Social Work, University of Southern California, Los Angeles, CA, United States; Department of Population and Public Health Sciences, University of Southern California, Los Angeles, CA, United States; Department of Population and Public Health Sciences, University of Southern California, Los Angeles, CA, United States; Department of Nutrition and Food Studies, George Mason University, Fairfax, VA, United States

**Keywords:** adolescents, stress, weight management, health behavior

## Abstract

**Objective:**

Adolescence is a critical period where growing autonomy over food choices shapes long-term health behaviors. While stress and dietary restriction are linked to eating behavior, prior research has examined trait-level stress, leaving momentary processes less understood. This study investigated associations between momentary perceived stress and eating behaviors, with dietary restriction tested as a moderator.

**Methods:**

Participants (*N* = 74 ages 14–17) from Los Angeles County completed a 10-day ecological momentary assessment protocol that included three to seven random smartphone prompts per day and initiated additional prompts during eating episodes. Perceived stress and dietary restriction were assessed at random prompts, while eating behaviors were assessed during eating-episode prompts. Eating behavior outcomes included dysregulated eating (loss of control eating and overeating) and food consumed (pastries/sweets and fruits/vegetables). Time-lagged generalized linear mixed models tested within- and between-subjects associations.

**Results:**

Neither within-subject perceived stress nor its interaction with restriction significantly predicted outcomes. No associations were found for predicting fruit and vegetable nor pastry/sweet consumption. However, in the main effect model, greater within- and between-subjects dietary restriction predicted subsequent loss of control eating, and higher between-subjects restriction predicted increased pastry/sweet intake.

**Conclusions:**

The findings highlight dietary restriction as a key acute predictor of adolescents’ loss of control eating, whereas momentary perceived stress did not emerge as a precipitant. Findings underscore the importance of screening for restrictive eating patterns and suggest that pediatricians and caregivers should monitor adolescents at risk. Future research should refine momentary stress assessment and test potential moderators of associations.

Adolescence is a key period in life for forming eating behaviors, as patterns established during this stage often persist as individuals become older and navigate increasing autonomy (Mikkilä et al., 2005). During adolescence, teenagers begin to have more control of their daily food choices compared to younger children (Bassett et al., 2008; Craigie et al., 2011; Sawyer et al., 2012; Spear & Kulbok, 2004). However, overweight and obesity are growing problems among adolescents in the United States. In fact, the prevalence of obesity among children and adolescents is 19.7% in the United States (Centers for Disease Control [CDC], 2024). The high prevalence of this issue is concerning, as having obesity during adolescence strongly predicts obesity in adulthood (Wang et al., 2008). Dietary patterns play a central role in this risk. Overconsuming energy-dense, high-fat, and low-fiber food during childhood, such as excess sugar intake and too little fruit and vegetable intake, has been consistently linked to greater risks of obesity, metabolic dysfunction, cardiovascular disease, inflammation, and several chronic conditions (Ambrosini, 2014; Angelino et al., 2019; Carboni et al., 2023; Dong et al., 2015; Field, 2017; Gillespie et al., 2023; Huang et al., 2023; Ledoux et al., 2011; Veronese et al., 2018). Similarly, behaviors such as dysregulated eating (i.e., binge eating, overeating, loss of control eating) are predictors of future obesity and weight gain (Field, 2017). Considering the impact of dietary intake and eating behaviors on development and chronic health conditions, it is important to explore risk factors for developing unhealthy eating patterns during adolescence.

## Stress and eating behavior

Stress influences individuals’ eating behaviors through various psychobiological processes. Stress elevates ghrelin and glucocorticoid (i.e., cortisol) levels, which in turn stimulate appetite and drive the desire for food intake (Bouillon-Minois et al., 2021; Sominsky & Spencer, 2014; Wren et al., 2001). In turn, consuming carbohydrate- and fat-dense foods increases the level of serotonin, a neurotransmitter that helps relieve stress and regulate mood, creating a short-term reward loop that reinforces the consumption of these foods (Dallman, 2010; Erlanson-Albertsson, 2005). In adults and adolescents, existing studies have reported that exposure to stress affects types of food consumed by decreasing consumption of healthy food, such as fruit and vegetables, and increasing intake of palatable, high-calorie food and between-meal snacks, even when a sense of hunger is absent (Dicker-Oren et al., 2024; Habhab et al., 2009; Lemmens et al., 2011; O’Connor et al., 2008; Oliver et al., 2000; Tryon et al., 2013). However, the relationship between stress and dysregulated eating is more complicated and unclear, with evidence from diverse adult samples showing that stress can either increase or decrease dysregulated eating (Azarbad et al., 2010; Freeman & Gil, 2004; Freizinger et al., 2022; Groesz et al., 2012; Hill et al., 2018; Lim et al., 2021; Rosenbaum & White, 2015; Torres & Nowson, 2007; Woods et al., 2010).

Importantly, previous studies have primarily adopted a cross-sectional design and examined adult samples, limiting their capacity in establishing causal or time-ordered relationships between stress and eating in youth (Azarbad et al., 2010; Freeman & Gil, 2004; Freizinger et al., 2022; Groesz et al., 2012; Hill et al., 2018; Rosenbaum & White, 2015; Woods et al., 2010). Additionally, most studies thus far have examined between-subject (i.e., trait) differences in stress as predictors of eating behaviors (Azarbad et al., 2010; Freeman & Gil, 2004; Groesz et al., 2012; Hill et al., 2018; Lim et al., 2021; Rosenbaum & White, 2015; Torres & Nowson, 2007; Woods et al., 2010). This fails to elucidate within-subject (i.e., state) differences. In other words, do short-term changes in momentary stress levels affect subsequent eating behaviors (e.g., within the next few hours) within an individual? Although an experimental study of adolescents found that cortisol reactivity to a stress induction, but not subjective stress reactivity, was associated with greater self-reported emotional eating (i.e., a form of dysregulated eating), no stress variables were associated with objectively measured caloric intake (Sato et al., 2023). This provides some support for a state association between physiological stress and eating behavior, but experimental work may lack generalizability to real-world eating behaviors.

### The role of dietary restriction in the association of stress and eating behavior

Dietary restriction may play an important role in predicting adolescents’ eating behaviors and may moderate the association of stress and eating behaviors. Dietary restriction refers to behavioral efforts individuals make to eat less, for the purpose of controlling their body weight (Svaldi et al., 2012). Restraint theory suggests that dietary restriction (e.g., meal skipping, avoiding eating) and restraint (e.g., cognitive attempts to eat less) may result in increased food intake, as constant inhibition of physiological signals for eating leads to preoccupation with food, thereby causing dysregulated eating and unhealthy food choices (Herman & Mack, 1975; Watson & Le Pelley, 2021). Expanding on restraint theory, others have posited that self-control used for dietary restraint draws on limited mental resources, which can be depleted when conducting cognitively taxing tasks (i.e., processing stress) (Loth et al., 2016; Svaldi et al., 2012). Together, these perspectives suggest that dietary restriction may not only fail to buffer against stress-induced eating behaviors but could, in fact, exacerbate them by increasing vulnerability to dysregulation when individuals are under stress.

When faced with stressors, individuals engaging in restrictive eating may have to shift their cognitive resources to handle the stressful emotions. As a result, they may become less able to suppress food intake or may use eating as a way to replenish depleted cognitive resources, which may lead to less suppression of food intake. As a result, individuals engaging in more dietary restrictions may be more likely to engage in dysregulated eating (e.g., loss of control eating, overeat, and not being able to stop eating) and make more unhealthy food choices when stressed (Loth et al., 2016). Scholars have attempted to test this proposition as a trait and found that restraint contributes to increased food craving and dysregulated eating, particularly when combined with other trait-level eating characteristics, particularly disinhibited eating among adult female samples (Habhab et al., 2009; Haynes et al., 2003; Linardon, 2018; Svaldi et al., 2012; Wallis & Hetherington, 2004; Wegner et al., 1993). When stress is taken into consideration, findings are mixed. For instance, some experimental studies have found that individuals with elevated dietary restraint consume more calories and greater amounts of palatable food when performing stress-inducing tasks, whereas others have found no such interaction (Habhab et al., 2009; Haynes et al., 2003; Roemmich et al., 2011). However, research has yet to test momentary changes in dietary restriction as a time-varying moderator of the within-subject association between stress and eating behavior in adolescents, which represents a more precise test of the proposed cognitive depletion model of binge eating (Loth et al., 2016).

### Importance of the adolescent development period

It is important to test a model of stress, dietary restriction, and eating behavior during adolescence, as this is a critical developmental period for psychosocial, cognitive, and behavioral changes (Amirkhan & Auyeung, 2007; Eiland & Romeo, 2013; Ragelienė, 2016; Spear & Kulbok, 2004). Understanding the impact of stress during this stage is crucial as the brain is more sensitive to stress-induced hormones compared to adults’ brains (Lee et al., 2003). Stress exposure during adolescence may generate heightened reactivity and hence disrupt brain reorganization and growth (Romeo, 2013). Consistently, studies have demonstrated that exposure to stressors during adolescence can affect brain functioning and increase susceptibility to dysregulated behaviors (Eiland & Romeo, 2013; Ganzel et al., 2013; Isgor et al., 2004; Oztan et al., 2011; Romeo, 2013). In addition, dietary restriction is a novel behavior during adolescence as it often emerges through the increasing importance of body image and peer influence as well as the emergence of social comparison behavior (D’Adamo et al., 2023; National Academies of Sciences, Engineering, and Medicine, 2019; Ragelienė, 2016; Stice et al., 2000). Therefore, adolescents may be particularly vulnerable to dysregulated eating after dietary restriction, especially when trying to regulate heightened stress. Altogether, heightened stress reactivity and novel eating-related decision-making in adolescence substantiate adolescence as a key period for studying stress-motivated eating behaviors and the potential maladaptive impact of dietary restriction.

## Ecological momentary assessment of stress, restriction, and eating behaviors

Ecological momentary assessment (EMA) is a well-suited method to test within-day associations of stress, dietary restriction, and eating behaviors in adolescents. EMA allows researchers to capture individuals’ momentary changes in psychological constructs over a short period of time and has been used to enhance understanding of how within-person fluctuations in various states affect one’s behavior (Trull & Ebner-Priemer, 2013). By using EMA, we can examine the within-person associations between perceived stress and eating behavior in adolescents’ daily-life environment and minimize recall biases inherent in retrospective study designs, regardless of being cross-sectional or longitudinal (Trull & Ebner-Priemer, 2013).

Existing studies with samples have found evidence that momentary stress may increase maladaptive eating patterns such as dysregulated eating and consumption of unhealthy food, especially sweets and fast food (Dicker-Oren et al., 2024; Leow et al., 2021; Reichenberger et al., 2021; Smith et al., 2021). Similarly, some prior research has shown that acute dietary restriction predicts subsequent binge-eating symptoms in adults and adolescents (D’Adamo et al., 2023; Zunker et al., 2011). However, less is known about how momentary stress relates to dysregulated eating and food intake in adolescents (Mason et al., 2020), including the interactive association of momentary stress with acute dietary restriction.

## The current study

The current study explored relationships between- and within-subjects’ perceived stress and subsequent eating behaviors including dysregulated eating (i.e., overeating and loss of control eating) and food intake (i.e., pastries/sweets and fruit/vegetable intake) among adolescents. We also examined whether between- and within-subjects dietary restriction moderated the associations of perceived stress with eating behaviors. Specifically, this study explored the following hypotheses: (1) higher between- and within-subjects perceived stress scores will prospectively predict more dysregulated eating, greater likelihood of pastry/sweet intake, and lower likelihood of fruit and vegetable intake at the next reported eating episode; (2) higher between- and within-subjects dietary restriction will prospectively predict more dysregulated eating, greater likelihood of pastry/sweet intake, and lower likelihood of fruit and vegetable intake at the next reported eating episode; and (3) elevated dietary restriction will augment the within- and between-subject relationship between perceived stress and dysregulated eating, greater likelihood of pastry/sweet intake, and lower likelihood of fruit and vegetable intake.

## Methods

### Study design and data

**Figure 1:**
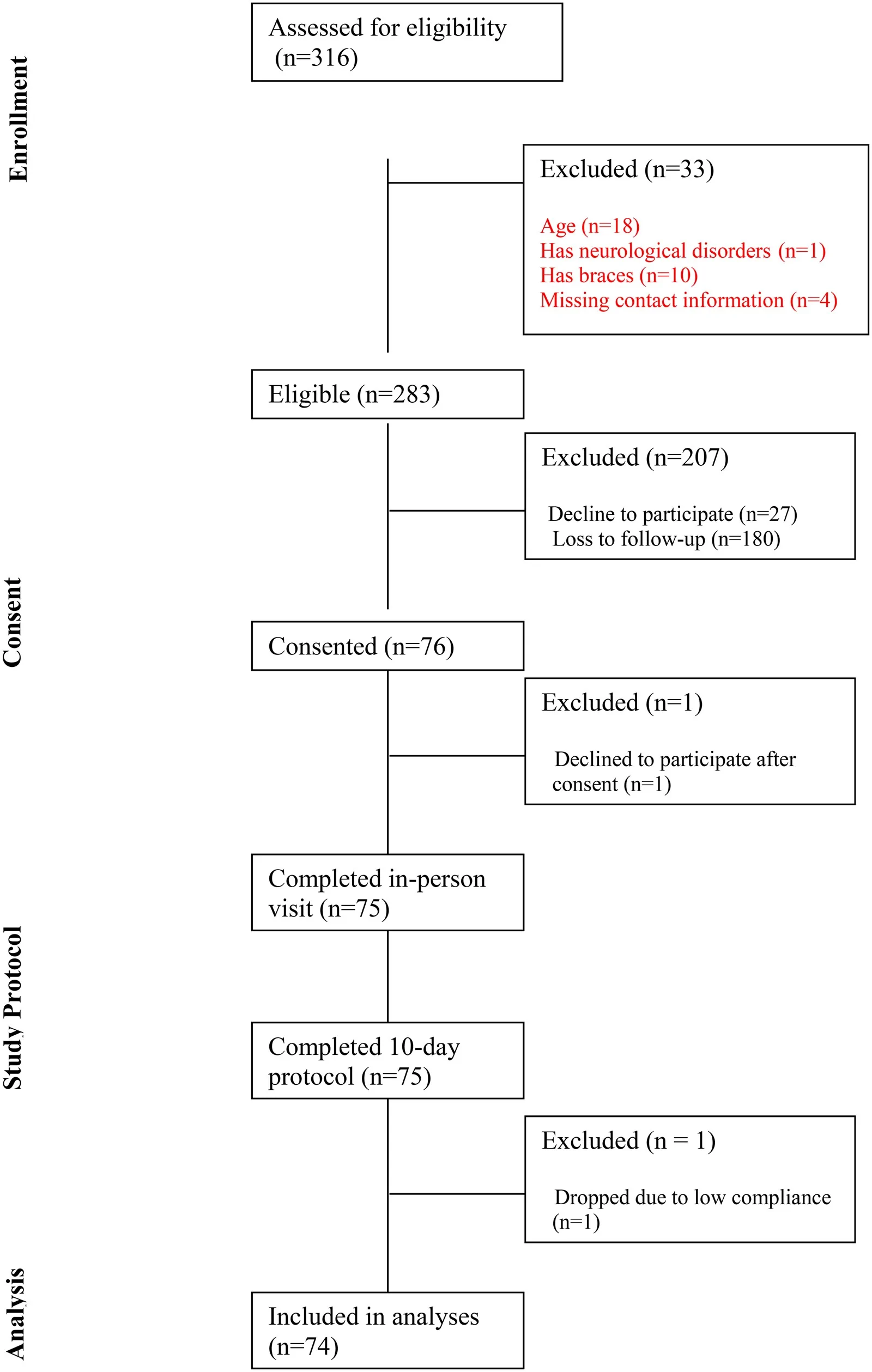
CONSORT flow diagram of participant recruitment and study participation.

This study conducted secondary data analyses of data from the Brain Influences on Teens’ Eating study. Prior papers from this dataset have examined the psychometric properties of the EMA dysregulated eating measure as well as momentary impulsivity as a predictor of dysregulated eating (Luo et al., 2025; Mason et al., 2025). The complete dataset is available on request. All study procedures were conducted in accordance with the ethical standards of the USC Institutional Review Board, which reviewed and approved the study protocol (IRB approval number: HS-20-00197; approval date: 3/5/2020). From October 2022 to September 2024, participants were recruited from a previous research study participant list and community and social media advertising. To be eligible for the study, individuals had to be between 14 and 17 years old and own a cellphone. Adolescents were excluded from the study if they had any history of head trauma or neurobiological disorders or were pregnant/breastfeeding. Research assistants contacted potential participants via phone. Eligible participants were invited to the study, and 75 teenagers were included in the study in total. One participant dropped out due to low compliance with the EMA protocol, leaving 74 eligible participants in the final data set. Caregivers provided written informed consent, and adolescents provided verbal and written assent to participate. Figure 1 presents a detailed recruitment flow chart.

The Brain Influences on Teens’ Eating study consisted of two phases: (1) one in-person clinic visit and (2) a 1-day consecutive EMA period in their natural environment. In the initial clinic visit, participants signed informed consent forms and completed self-report questionnaires. Participants’ demographic information, including race and ethnicity, biological sex, sexual orientation, and age, was collected in the self-reported questionnaires. Anthropometric measurements, including their height and weight, were collected and converted into body mass index z-score (BMIz) using the CDC Growth Charts corresponding to participants’ age and biological sex (CDC, 2024). Additionally, they received EMA training on their phones before starting the 10-day EMA protocol. Participants were compensated $120 for their in-person visit and a $20 compliance bonus if they complete 80% or more of the EMA prompts.

During the 10-day EMA period, participants completed surveys using the LifeData EMA application on their smartphones. Two types of EMA measures were collected, including random prompts and eating prompts. Notifications were pushed to participants’ cellphones between 3:30 and 9:30 p.m. on weekdays and 9:00 am and 10:00 p.m. on weekend days. Random prompt involved signal-contingent reporting, where participants were prompted to complete the survey three times during weekdays and seven times during weekends. Whereas the eating prompt involved event-contingent reporting and was completed after engaging in an eating episode. Participants were instructed that an eating episode referred to anytime they consumed any food, including snacks and meals, regardless of the amount they consumed. They were also able to record missed eating episodes during signal-contingent recordings. Participants were instructed not to complete surveys if they could not do so safely; however, they were encouraged to complete the entry when they were able to do so. Research staff conducted check-ins with participants who were not meeting EMA compliance to address any questions or concerns they might have.

### Measures

#### Perceived stress

Two items, adapted from the Stress in Children Scale (Osika et al., 2007), were used to assess perceived stress. The two items were “I can manage all the things I have to do right now” and “Things are working out as I have planned right now.” Each item was rated on a 4-point scale, ranging from 1 (*not at all*) to 4 (*extremely*). Participants were prompted to respond to this scale in EMA random prompts. Items were reverse coded such that higher values indicated more perceived stress than individuals could handle. Finally, the two items were averaged to create a composite perceived stress score for each prompt. These items have been used in prior EMA research of children (Do et al., 2021; McAlister et al., 2024); although, one of the prior studies used a dichotomous (yes/no) response option rather than a rating scale (Do et al., 2021). In a study of 8-12-year-old children, the composite score of the two perceived stress EMA items, which used a dichotomous response option for items, was positively correlated with scores on the Stress in Children Scale, providing some evidence of concurrent validity (Naya et al., 2021).

#### Dietary restriction

A three-item checklist was used to measure dietary restriction behaviors, which have been used in prior EMA research in adults (Mason et al., 2023). Participants indicated which of the following behaviors they engaged in, such as “Avoided eating in an attempt to control my weight,” “Skipped a meal (or meals) in an effort to control my weight”, and “Refused food or drink offered because I was concerned about my weight.” Response options were 0 (*no*) and 1 (*yes*). Participants were prompted to respond to this scale in EMA random prompts. A sum score was created, indicating the number of restrictive behaviors engaged in at each prompt.

#### Dysregulated eating

Five items were used to measure dysregulated eating (Goldschmidt et al., 2018). These include “While you were eating, to what extent did you feel a sense of loss of control”; “While you were eating, to what extent did you feel you could not stop eating once you started”; “While you were eating, to what extent did you feel disconnected (e.g., numb, zoned out, on autopilot)”; “To what extent do you feel that you overate”; and “To what extent do you think that others would consider what you ate to be an unusual or excessive amount of food?” Each item is rated on a 5-point scale from 1 (*not at all*) to 5 (*extremely*). Participants were prompted to respond to this scale in EMA eating prompts. Our own analyses showed the five items are best represented as two factors (i.e., loss of control eating and overeating), and factors show construct validity with associations between overeating and loss of control eating with unhealthy food intake (Luo et al., 2025). As such, we created loss of control eating and overeating subscales, with higher scores indicating more dysregulated eating.

#### Food choice

Prior EMA work developed a food item checklist to capture daily food choice (Bruening et al., 2016). Two types of food, fruit and vegetables (including salads) and cookies/sweetened baked goods/candy/frozen desserts, were considered in the study. A similar item was used in prior EMA research with youth (O’Connor et al., 2018), which showed good concordance with 24-h recall data. During eating prompts, participants were asked whether they had consumed either of these food types in their current eating episode, with response options of 0 (*no*) and 1 (*yes*). Each response was treated as a dichotomous outcome in the subsequent analysis. Food item checklists have been completed in EMA by children in prior research (Mason et al., 2019). For clarity and succinctness in reporting the results, cookies/sweetened baked goods/candy/frozen desserts are collectively operationalized as “pastries/sweets” in this paper.

### Statistical analysis

All data analysis was conducted in STATA 18.0 (StataCorp, 2023). Descriptive statistics analyses were run on participants’ demographic characteristics, anthropometric measurements, perceived stress, dietary restriction, food choice, and dysregulated eating. For predictors (i.e., perceived stress and dietary restriction), effects were disaggregated into within- and between-subjects effects (Curran & Bauer, 2011). The between-subjects effects were created by computing an aggregated mean of each variable for each participant for all their entries throughout the EMA protocol. The within-subject effects were centered by deducting the aggregate mean from the observed value at each EMA prompt. To examine whether stress and dietary restriction predict eating behaviors at the next time point, time-lagged effects were accounted for by shifting the prior observation forward to align with the current eating behavior variables. The nearest recorded values for prior stress and dietary restriction were used to predict current eating behavior. Two interaction terms were created by multiplying the values of perceived stress and dietary restriction: one term for the within-subject level of perceived stress and dietary restriction and the other one for the between-subject level perceived stress and dietary restriction.

Before running the primary models, covariates, including sex, age, window of data collection (whether collected during school time or summer break), and BMIz, were screened and were included in the primary analyses if related to the dependent variables at *p* = .05. The window of data collection was operationalized as a binary variable, coded as 1 for data collected between June 15 and August 15 (as an approximate indicator of summer break) and 0 otherwise. Generalized linear mixed models (GLMMs) were employed to examine the associations of within- and between-subjects stress and dietary restriction and the two-way interaction of stress and dietary restriction with dysregulated eating and food choice. For dysregulated eating, a gamma log link function was chosen because dysregulated eating was continuous and positively skewed. For the dichotomous food choice variables, logistic link functions were used, as outcomes were binary. The same set of covariates were included in the analyses. Effect parameters were converted into odds ratios.

## Results

Table 1 presents descriptive statistics of demographic characteristics. The sample had a mean age of 15.7 years old and consisted of slightly more females than males. Most participants identified as heterosexual, while a smaller proportion identified as bisexual or gay/lesbian, with some uncertainty or other identities reported. The sample was racially diverse, with White and Asian participants representing the largest groups. Additionally, 38.7% of participants identified as Hispanic. About 15% of the sample met criteria for overweight (BMI percentile between 85th and 95th for their age and gender) and 19% met criteria for obesity (BMI percentile above 95th).

**Table 1:** Distribution of demographic characteristics among the total sample (*N* = 75).

Demographic characteristic	*M* (*SD*) or *N* (%)
**Age (years)**	15.7 (1.14)
**BMI**	24.2 (5.6)
**BMIz**	0.704 (1.08)
**Sex (%^a^)**	
**Male**	28 (37.3%)
**Female**	47 (62.7%)
**Gender identity (%)**	
**Male**	29 (38.7%)
**Female**	44 (58.7%)
**Do not identify as either male of female**	1 (1.3%)
**Not sure yet**	1 (1.3%)
**Sexual orientation (%)**	
**Straight**	52 (69.3%)
**Gay or lesbian**	3 (4%)
**Bisexual**	12 (16%)
**Not sure**	5 (6.7%)
**Other**	3 (4%)
**Hispanic (%)**	
**Yes**	29 (38.7%)
**No**	46 (61.3%)
**Race (%)**	
**Asian**	20 (26.7%)
**Black or African American**	5 (6.7%)
**White**	29 (38.6%)
**Other**	20 (26.7%)^b^
**Not reported**	1 (1.3%)

a Percentages have been rounded to one decimal place.

b The “Other” race category primarily reflected Hispanic-identifying participants (*n* = 18).

Across EMA, there were 2,162 random prompts and 856 event-contingent completed. Among all the responses, there were 1,355 eating episodes reported. The mean EMA compliance for random prompts was 67.48% (*SD* = 23.65%; range: 10.35%–97.83%). A total of 31 participants received a $20 bonus for achieving a compliance rate exceeding 80%. The average interval between the current eating episode and prior stress and dietary restriction observation was 2.06 h (*SD* = 0.59; range: 1.28–5.71). None of the demographic factors correlated with EMA compliance. Table 2 shows the descriptive statistics for the EMA study variables. Participants reported relatively elevated perceived stress levels throughout the EMA protocol, with the observed mean slightly exceeding the scale’s median. Overeating, loss of control eating, and dietary restriction behavior were uncommon yet showed variability across EMA. Among all the responses that recorded food choice data, about a quarter reported consuming either fruit/vegetables or pastries/sweets.

**Table 2:** Descriptives of key variables of interest.

Variables	*M* (*SD*) or *N*(%)
**Perceived stress^a^**	2.73 (0.81)
**Dietary restriction**	0.124 (0.502)
**0 restriction behavior (%^b^)**	1918 (92.9%)
**1 restriction behavior (%)**	76 (3.7%)
**2 restriction behaviors (%)**	31 (1.5%)
**3 restriction behaviors (%)**	39 (1.9%)
**Dysregulated eating**	
**Loss of control eating**^c^	1.43 (0.716)
**Overeating ^d^**	1.48 (0.771)
**Fruit and vegetable (yes)**	350 (25.8%)
**Pastries/sweets (yes)**	292 (21.6%)

a Possible range is 1–4.

b Percentages have been rounded to one decimal place.

c Possible range is 1–5.

d Possible range is 1–5.

Tables 3 and 4 present the detailed statistics of the main effect models and interaction models, respectively. Covariates included in the GLMMs were age, sex, and BMIz, as they were significant in the univariable covariate selection analyses. The window of data collection variable was not significantly associated with any outcome and was therefore excluded from subsequent analyses. Only BMIz remained significantly associated with overeating in both models. The first hypothesis was not supported. Only between-subject stress was negatively correlated with overeating; that is, adolescents who reported higher stress across EMA engaged in less overeating overall. Therefore, the second hypothesis was partially supported. Within-subjects dietary restriction significantly predicted loss of control eating on both within- and between-individual levels. The within-subjects effect shows that greater dietary restriction compared to one’s own average predicts an increased subsequent loss of control eating in the next few hours. The between-subjects effect shows that adolescents who engaged in more dietary restriction on average, compared to others, had more loss of control eating. For food choice outcome, between-subjects dietary restriction was associated with a higher frequency of pastry/sweet consumption, indicating that adolescents with more restriction, compared to others, were more likely to eat pastries/sweets than those reporting less average restriction. The third hypothesis was not supported, as the interaction terms between stress and restriction on both levels were not significant for any outcome. As a sensitivity analysis, we ran a three-way interaction model with perceived stress, dietary restriction, and the time interval between the lagged prompt and the eating episode. There was no significant impact of time.

**Table 3:** GLMM of main effects of perceived stress and dietary restriction on loss of control eating, overeating, and food choice.

	Loss of control eating		Overeating		Pastries/sweets		Fruit and vegetable	
Variables	Est.	*SE*	Est.	*SE*	OR	*SE*	OR	*SE*
**Sex (ref: male)**	0.061	0.075	0.027	0.069	0.674	0.273	2.39*	0.949
**Age**	0.042	0.031	0.044	0.029	1.28	0.223	.972	0.158
**BMIz**	0.040	0.033	0.061*	0.030	1.19	0.202	1.05	0.178
**Within-subject stress**	−0.012	0.019	−0.029	0.028	1.23	0.250	1.02	0.189
**Between-subject perceived stress**	−0.082	0.061	−0.129	0.058	1.60	0.558	1.77	0.581
**Within-subject dietary restriction**	0.078	0.025	0.014	0.037	1.03	0.265	1.08	0.262
**Between-subject dietary restriction**	0.434	0.165	0.281	0.151	6.53	5.44	.873	0.699
**Pseudo *R*²**	0.046		0.020		0.019		0.010	

* *p*> 0.05.

**Table 4:** GLMM of moderation effects of perceived stress and dietary restriction on loss of control eating, overeating, and food choice.

	Loss of control eating		Overeating		Pastries/sweets		Fruit and vegetable	
Variables	Est.	*SE*	Est.	*SE*	OR	*SE*	OR	*SE*
**Sex (ref: male)**	0.054	0.075	0.018	0.069	0.666	0.271	2.38*	0.952
**Age**	0.043	0.031	0.047	0.028	1.28	0.223	0.972	0.158
**BMIz**	0.041	0.033	0.063*	0.029	1.19	0.203	1.05	0.179
**Within-subject perceived stress**	−0.013	0.019	−0.029	0.028	1.23	0.250	1.03	0.189
**Between-subject perceived stress**	−0.099	0.624	−0.161	0.061	1.54	0.580	1.75	0.618
**Within-subject dietary restriction**	0.058	0.029	0.019	0.043	1.05	0.295	1.07	0.286
**Between-subject dietary restriction**	−0.625	1.26	−1.26	1.12	1.43	8.84	0.526	3.25
Within-subject perceived stress * dietary restriction	0.052	0.036	−0.008	0.051	0.958	0.375	1.04	0.350
Between-subject perceived stress * dietary restriction	0.521	0.624	0.767	0.552	2.14	6.54	1.28	3.89
**Pseudo *R*²**	0.051		0.022		0.019		0.013	

* *p* > .05,

** *p* > .01,

*** *p* > .001.

## Discussion

Understanding adolescents’ eating behaviors is critical as it is a prominent stage for forming eating habits along with increased autonomy and choices, and it is becoming increasingly more important given the rising prevalence of obesity and poor diet in the United States (Bassett et al., 2008; CDC, 2024; Mikkilä et al., 2005; Spear & Kulbok, 2004). This investigation examined the dynamic relationships between stress, dietary restriction, and eating behavior outcomes among adolescents, with analyses showing mixed support for our hypotheses.

Our major finding is that state- and trait-level dietary restriction predicted increased loss of control eating at the subsequent eating prompt. This supports the restraint model of binge eating, which suggests that individuals engage in binge eating following behavioral restriction. Existing studies have found associations between dietary restraint and restriction and dysregulated eating among youth and adults, with the current study providing more support (D’Adamo et al., 2023; Neumark-Sztainer et al., 2007; Woods et al., 2010). Our results support the possibility that adolescents could be susceptible to a cycle of restriction and loss of control eating, whereby persistent efforts to reduce intake lead to subsequent greater consumption and then future attempts to restrict.

Within-subjects dietary restriction was not related to overeating or food choice, which suggests that restriction does not reliably predict the amount of food or type of food eaten at the subsequent eating episode. Yet, research has shown that eating episodes defined by higher loss of control are associated with overeating and consumption of unhealthy foods (Luo et al., 2025); therefore, loss of control may represent intake of foods that were being restricted. Between-subjects dietary restriction was associated with more pastries/sweets consumption. It may be that individuals who consume more sweet food in general are more likely to engage in restriction. For example, previous trait-level research found that individuals with elevated dietary restraint were more likely to consume palatable foods after suppressing cravings (Erskine & Georgiou, 2010; Zhang et al., 2021). Therefore, it may be important to account for other psychological factors in these associations, such as food cravings.

Contrary to our original hypotheses, we did not identify a relationship between within-subjects’ perceived stress and subsequent eating outcomes nor an interaction between stress and restriction in predicting eating behaviors. These findings diverge from some prior EMA studies, which have documented a positive within-subject association between stress and dysregulated eating, particularly in clinical samples of adults diagnosed with eating disorders (Freeman & Gil, 2004; Goldschmidt et al., 2014; Smyth et al., 2007). One potential explanation for this discrepancy lies in differences in sample characteristics. Our study focused on non-clinical adolescents who were not part of a clinical population, whereas previous research has predominantly investigated clinical adult populations. Individuals with eating disorders show learned expectancies that eating can serve as a coping mechanism (Hohlstein et al., 1998); adolescents without eating disorders may still be developing stress coping strategies and thus may vary in learned expectancies for how eating can relieve stress. It may be important to study various eating expectancies as a contributor to the stress and eating relationship, as has been done in prior research (e.g., Pearson et al., 2018). Moreover, adolescents typically have limited autonomy over food access due to financial and environmental constraints, such as reliance on caregivers for food and fewer opportunities to purchase food independently. These contextual factors may moderate the stress-eating relationship in adolescents.

Instead, a negative association between perceived stress and overeating emerged at the between-subject level, suggesting that adolescents who, on average, had higher stress than they could handle tended to report less overeating overall. Although many studies have documented a positive association between stress and binge eating, research has sometimes found the opposite pattern, that individuals experiencing higher stress consumed less food (Hill et al., 2022; Klatzkin et al., 2018; Sominsky & Spencer, 2014). For example, Nagy et al.(2019) found that those with high cortisol reactivity exhibited a significant decrease in caloric intake following a stressor. This aligns with physiological models in which stress triggers an increase in cortisol and leptin, temporarily suppressing appetite. Together, these findings indicate that stress does not uniformly promote overeating; for some individuals, heightened chronic stress could be associated with reduced overeating.

The null findings regarding the interaction between perceived stress and dietary restriction may similarly be understood in light of these developmental and contextual differences. The resource depletion model of self-control posits that stress, when combined with dietary restriction, can overwhelm self-control and promote disinhibited eating (Loth et al., 2016). But among adolescents, the capacity for dysregulated eating may be limited due to food availability, obscuring the effects of stress-restriction interactions. These constraints are further reflected in the relatively low base rates of overeating and loss of control eating in this non-clinical sample, with the predictors of dysregulated eating potentially being different for youth with significant baseline dysregulated eating or eating disorders. Additionally, the affect regulation model suggests that difficulties in managing negative emotions and maintaining goal-directed behaviors contribute to dysregulated eating, particularly in adult clinical populations (Kenny et al., 2017; Munsch et al., 2012). If perceived stress is not appraised in a way that meaningfully elevates negative emotions, the hypothesized interactive effect may not emerge.

Another plausible explanation for our findings concerns the way stress was measured. In the present study, the two items from the Perceived Stress Scale emphasized participants’ perceived ability to manage demands in the moment rather than the intensity of stressors. This measure likely captures the secondary appraisal process—how capable individuals feel in handling a given situation—rather than the intensity of the stressor itself (Lazarus & Folkman, 1984). In contrast, prior studies that found significant associations between stress and dysregulated eating often used measures that directly assessed emotional distress or perceived stress intensity, aligning more closely with primary appraisal processes (Goldschmidt et al., 2014; Smyth et al., 2007). Measures focused on stress intensity or emotional distress may capture aspects of the stress response that are more closely related to eating behavior than measures focused primarily on coping capacity and stress appraisal.

Related, Sato et al. (2023) found that chronic subjective stress and cortisol reactivity predicted higher self-reported emotional eating in a non-clinical sample of adolescents, whereas subjective stress reactivity was not associated with emotional eating or objectively measured caloric intake. These findings suggest that different dimensions of stress may show distinct relationships with eating outcomes, underscoring the importance of using multidimensional assessments of stress. Therefore, future studies may employ more comprehensive assessments of momentary stress that capture multiple dimensions of the stress response, including perceived stress intensity, stress appraisal, emotional distress, physiological indicators, and dynamic changes in stress over time. For example, in a study on adults with binge-eating disorder, stress was assessed by measuring the occurrence of stressful events and then categorizing its effects into stress reactivity (the difference between perceived stress at a stressed moment and the last non-stressed moment), stress recovery (the difference between perceived stress at a stressed moment and the following non-stressed moment), and stress pileup (the accumulation of stress over the previous 12 h); analyses showed that stress pileup significantly predicted increased binge-eating symptom severity (Smith et al., 2021). Taken together, these findings suggest a complex relationship between stress and dysregulated eating in adolescents, which requires a multidimensional assessment approach that captures both appraisal processes and physiological stress responses.

Although not all hypotheses were confirmed, several clinical implications stem from these results. First, we found that dietary restriction predicts loss of control eating in adolescents on both between- and within-individual levels. Pediatricians and caregivers should monitor adolescents for dietary restriction and provide early intervention to prevent dysregulated eating. Possible preventions for loss of control eating might include having families schedule regular meals and snacks as well as nutrition therapy. Second, given the null findings for stress, more research will be needed to determine the utility of stress interventions in preventing adolescent dysregulated eating. Lastly, BMIz remained significant in both models. Prior evidence has shown that stress, dietary restriction, and eating behaviors may operate differently across weight categories (Caso et al., 2020; Dallman, 2010), underscoring the need for future work to understand how stress and restriction impact eating in youth of different weight groups.

### Strengths and limitations

This study has several strengths, including the use of EMA to capture real-time eating behaviors, dietary restriction, and perceived stress among a community sample of adolescents, which reduced retrospective bias, improved ecological validity, and allowed for studying within-subjects processes. However, there are also limitations that need to be considered. When examining food types (i.e., pastries/sweets and fruits/vegetables), we did not assess portion size, which limited the assessment of the food type outcomes. For example, stress and restriction may predict over-consumption of specific food types. Also, while the two perceived stress items have been used in prior youth studies and were taken from a validated self-report questionnaire, there is limited data on the psychometric properties of the two-item Perceived Stress Scale. Future work is needed to develop more comprehensive EMA measures of stress in youth. Our compliance was slightly lower than the average EMA compliance rate of adolescents in a meta-analysis (Wen et al., 2017). This was observed despite the provision of additional monetary incentives for high compliance, suggesting that other incentive structures may be needed to substantially improve compliance in adolescents. In samples from non-clinical settings, higher prompt frequency has been linked to lower compliance, which may partly account for the reduced compliance observed in our study, given that participants received seven prompts on weekend days (Wen et al., 2017). Also, missing prompts, misreporting, or delayed reporting could have reduced the likelihood of capturing all eating episodes and items consumed. Because EMA relies on self-report, socially undesirable behaviors (e.g., dysregulated eating, pastries/sweets consumption) may have been underreported, and stressful events occurring outside of signal-contingent or random prompts may have been missed. The relatively low number of reported pastries/sweets and fruits/vegetables may reflect limitations of the measurement approach, including underreporting and/or lack of reporting food consumed before 3:30 p.m. on weekdays. Moreover, EMA designs cannot ensure that participants initiate an eating-episode prompt immediately after eating, and the actual lag between eating and reporting cannot be verified, which introduces additional uncertainty.

Measurement of dietary restriction may not include all types of restriction behaviors, such as avoiding specific food groups, prolonged fasting, and other types of food. Future studies may benefit from using a more comprehensive assessment of food types to avoid the need to estimate multiple separate models. Further, while all EMA items have been used previously, they have not all undergone rigorous psychometric evaluation in adolescents. Also, because this sample was neither population-based nor clinical, the generalizability of findings to broader adolescent populations may be limited. Future studies should consider more diverse samples, including clinical populations, and incorporate objective dietary measures to improve external validity. Finally, food access and parents’ feeding practices were not directly assessed in the present study, yet prior research identified that both may play important roles in shaping eating behaviors. Specifically, experiencing food insecurity has been associated with greater use of restrictive feeding practices by caregivers, and such restrictive practices have been linked to dysregulated eating patterns in children (Baxter et al., 2022; Birch et al., 2003). As adolescents gain greater autonomy over food choices, they may internalize caregiver feeding practices, which could influence eating behaviors independent of structural food access (Burnette et al., 2023). Taken together, these findings and the broader literature highlight the need to better examine the potential role of food access and parenting feeding patterns in future studies of stress, restriction, and youth eating.

## Conclusion

This study highlighted a dynamic relationship between dietary restriction and loss of control eating among adolescents in their natural environments but no effects for perceived stress. Also, the findings did not support stress and restriction as dual contributors to dysregulated eating or food choice as posited by resource depletion models (Loth et al., 2016). Future research with diverse clinical and community adolescent samples should continue examining within-subjects predictors of eating behaviors using multidimensional measures of stress to further clarify these relationships.
